# Claudin Family Participates in the Pathogenesis of Inflammatory Bowel Diseases and Colitis-Associated Colorectal Cancer

**DOI:** 10.3389/fimmu.2019.01441

**Published:** 2019-06-27

**Authors:** Liguo Zhu, Jing Han, Li Li, Ying Wang, Ying Li, Shenghong Zhang

**Affiliations:** Division of Gastroenterology, The First Affiliated Hospital, Sun Yat-sen University, Guangzhou, China

**Keywords:** inflammatory bowel diseases, claudin, intestinal permeability, colitis-associated colorectal cancer, protein family

## Abstract

Claudins are a multigene transmembrane protein family comprising at least 27 members. In gastrointestinal tract, claudins are mainly located in the intestinal epithelia; many types of claudins form a network of strands in tight junction plaques within the intercellular space of neighboring epithelial cells and build paracellular selective channels, while others act as signaling proteins and mediates cell behaviors. Claudin dysfunction may contribute to epithelial permeation disorder and multiple intestinal diseases. Over recent years, the importance of claudins in the pathogenesis of inflammatory bowel diseases (IBD) has gained focus and is being investigated. This review analyzes the expression pattern and regulatory mechanism of claudins based on existing evidence and elucidates the fact that claudin dysregulation correlates with increased intestinal permeability, sustained activation of inflammation, epithelial-to-mesenchymal transition (EMT), and tumor progression in IBD as well as consequent colitis-associated colorectal cancer (CAC), possibly shedding new light on further etiologic research and clinical treatments.

## Introduction

Claudins, a multigene transmembrane protein family comprising at least 27 members (1), reportedly contain four transmembrane (TM) helix domains, two extracellular loops (ECLs), a short N-terminus and a C-terminus (2). In gastrointestinal tract, claudins are mainly located in the intestinal epithelia; many species of claudins form a network of strands in tight junction (TJ) plaques within the intercellular space of neighboring epithelial cells and build paracellular selective channels (3–5), while the others act as signaling proteins and modulate cell behaviors (6). Consequently, claudins dysfunction that occurs in enterocytes may contribute to epithelial permeation disorder and multiple intestinal diseases, including inflammatory bowel diseases (IBD) (7, 8). IBD are a series of chronic multifactorial gastrointestinal inflammatory disorders that mainly include Crohn's disease (CD) and Ulcerative Colitis (UC), with rather high non-response rates and recurrence rates in clinical practice. Moreover, evidence regarding the correlation of IBD with colorectal cancers exists, as sustained extensive colitis is a known independent risk factor for colitis-associated colorectal cancer (CAC) (9, 10). However, owing to sophisticated etiology, the dynamics of IBD and consequent tumorigenesis remain obscure. As the roles of claudins have been gradually better understood in recent years, dysregulation of different claudin types may modulate barrier permeability as TJ proteins and impact tumor behaviors as signaling proteins, therefore participating in the pathogenesis of IBD and consequent tumorigenesis (11).

## Pathogenesis of IBD

**Figure 1:**
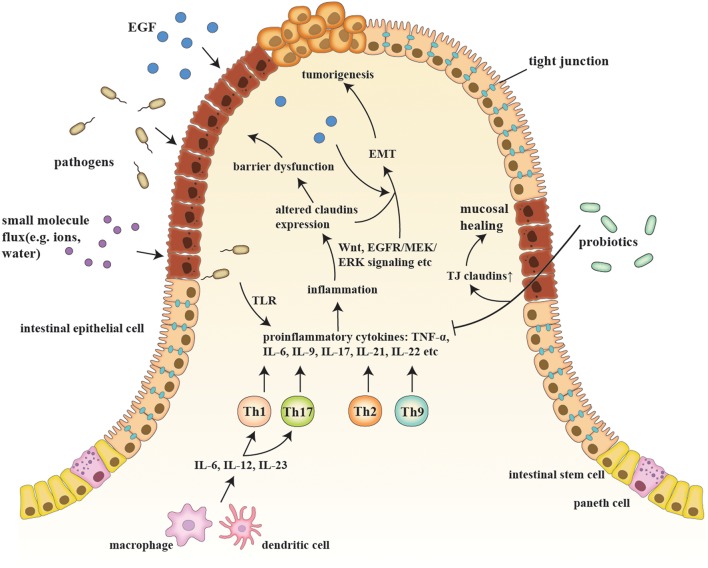
A schematic model of claudins in the pathogenesis of IBD and consequent tumorigenesis. Etiologic factors, such as a pathogenic breach and dysbiosis, engender an overactive inflammatory response by secreting proinflammatory cytokines (e.g., IL-6, IL-9, and IL-23) and generating proinflammatory T cells (e.g., Th1, Th2, Th9, and Th17), which also changes claudin expression via multiple pathways, such as Wnt and MEK/ERK signaling. Claudin dysregulation directly leads to impaired barrier function and luminal bacterial leakage inversely exacerbates inflammation, while EGF influx contributes to tumorigenesis. EMT and tumorigenesis are results of sustained activation of signaling pathways that modulate cell growth and migration, in which claudins act as signaling proteins and modulate aggressive tumor behaviors. Probiotics, on the other hand, recover epithelial claudin expression, and therefore promote mucosal healing.

IBD are a series of chronic gastrointestinal inflammatory disorders, among which CD and UC are the most prevalent and well-studied (12). The sophisticated etiology of IBD is described in the following sub-sections, along with the relationship of IBD to claudins (Figure 1).

### Genetic Variants Related to Disease Susceptibility

Over 200 genetic risk loci are associated with IBD in modulating barrier homeostasis, intestinal epithelial renewal, defense against microbes, and innate/adaptive immune regulation, among other things (13, 14). In a study on twins discordant for CD, healthy co-twins exhibited barrier function changes similar to their CD co-twins (e.g., increased small molecule permeability and reduced claudin-5 expression) when compared with non-related controls, indicating that genetic predisposition may be an important etiologic factor in IBD onset (15). Some gene variants may increase IBD predisposition by modulating TJ proteins because they take on the dual role of sealing proteins and signaling proteins. *NOD2* mutations engender anti-biotic α-defensin reduction and altered composition of TJ proteins (e.g., claudin-1, claudin-2, occluding, and Zonula Occludens-1), resulting in higher susceptibility to luminal bacterial infection in CD patients (16) and exacerbation of inflammation via interleukin-8 (IL-8)/nuclear factor-κB (NF-κB) activation in epithelia as well as Toll-like receptor-2(TLR2)-dependent interferon-γ (IFN-γ) upregulation in antigen-presenting cells (17, 18). Similarly, *SHANK3* mutations downregulate ZO-1 by inhibiting protein kinase Cε (PKCε), thus engendering bacterial paracellular influx and increasing propensity to inflammation in CD patients (19), which is also likely a mechanism of claudins dysregulation in IBD onset that awaits further analysis.

### Aberrant Responses of Innate and Adaptive Immunity

At their core, IBD are a series of autoimmune diseases, and aberrant immune responses may contribute to IBD in sophisticated ways by involving both innate and adaptive immune mechanisms (20). The function of T cells and relevant cytokines is well-studied and considered important in the pathogenesis of IBD. Under physiological conditions, T_reg_ cells and macrophages secrete transforming growth factor-β (TGF-β) and IL-10 to induce immunotolerance (21). However, under pathological conditions such as infections, the upregulated proinflammatory cytokines (e.g., IL-1, IL-6, IL-12, and IL-23) and generation of Th1, Th2, Th9, and Th17 cells, along with the activation of other immune cells (e.g., neutrophils, NK cells), cooperatively construct an elaborate network triggering IBD (21). For example, Th9 subset exacerbates murine experimental colitis, increases bacteria permeability and impairs wound healing by secreting IL-9 and upregulating pore-forming claudin-2 (22). From a viewpoint of clinical applications, except for anti-tumor necrosis factor (TNF)-α agents, the efficacy and safety of other inhibitory agents against the participating immune cells and cytokines, such as anti-integrin and anti-IL-23 agents, still need to be examined through clinical trials (23, 24).

### Dysfunction of Mucosal Barrier

TJ-dependent paracellular passages manage the exchange of paracellular substances between the intestinal lumen and internal environment, thereby playing a role in the balancing of nutrient absorption and waste secretion as well as defense mechanisms against pathogens. In accordance with the well-acknowledged roles of claudins in forming TJs and selective channels, claudins may participate in both types of transepithelial paracellular leakage (25): proinflammatory cytokines-induced small molecule (e.g., ions and mannitol) channel disruption and cell detachment-induced large molecule (e.g., epidermal growth factor, EGF) leakage (26). Thus, as barrier-forming proteins, dysregulated expression and redistribution of claudins may lead to increased intestinal permeability, susceptibility to gut infection and bowel symptoms of IBD patients (27–29).

### Imbalance of Intestinal Microbial Colonization

Dysbiosis of microbiota may influence mucosal homeostasis, immune response, nutrient uptake, and vitamin production with altered metagenome and perturbed microbial metabolism, finally contributing to IBD (30, 31). For example, adherent-invasive *Escherichia coli*, a prevalent pathogen in chronic and early recurrent ileal lesions in CD patients (32), interferes with host immune responses by surviving macrophage phagocytosis, inducing neutrophil autophagy, promoting Th17 differentiation, and upregulating proinflammatory cytokines TNF-α and IL-6 (33–35). Probiotics, such as *Bacillus subtilis* and *Bifidobacterium longum*, on the other hand, alleviate inflammation and repair barrier function by downregulating proinflammatory cytokines (e.g., IL-17, IL-23, and TNF-α) and upregulating TJ proteins (e.g., claudin-1, occluding, and ZO-1) in colitis mice models (36–38).

### Individual and Environmental Risk Factors

Based on existing epidemiologic research, industrialization is regarded as a significant risk factor for IBD because its incidence rate in western countries has increased rapidly since the 1950s and has remained high (39, 40), while the low incidence rate in newly industrialized countries has steadily begun to rise in the twenty-first century (41). Age may also be a risk factor for CD and UC, because the first diagnostic peak appears at 20–30 years in CD and at 30–40 years in UC, along with a possible second peak at 60–70 years (42). Other risk factors, including smoking, appendectomy, dietary habits, antibiotic use, and childhood exposure, may also be relevant to the pathogenesis of IBD (43, 44). Interestingly, second-hand smoking engenders claudin-3 and ZO-2 upregulation in murine large intestines possibly by inhibiting NF-κB signaling and AMP-activated protein kinase (AMPK), but also increases oxidative stress by activating c-Jun N-terminal kinase (JNK) and p38 mitogen-activated protein kinase (MAPK) signaling, indicating the complicated role of smoking in modulating inflammation (45).

## Tumorigenesis of IBD

The sophisticated pathogenesis of CAC is associated with genomic alterations, inflammation-induced aberrant immune response, and alterations in bowel microbiota, etc. (46). Sequencing analysis has revealed that genomic alterations in CAC are significantly different from sporadic colorectal cancer with regard to a higher frequency of *TP53, MYC*, and *IDH1* mutations, along with lower frequency of *APC* mutations (47). Moreover, as colitis-associated intestinal barrier leak allows for the paracellular influx of luminal growth factors, EGF triggers sustained activation of Ras/Raf/mitogen-activated ERK kinase (MEK)/extracellular signal-regulated kinase (ERK) signaling, phosphatidylinositol-3-kinase (PI3K)/ protein kinase B (Akt) signaling as well as signal transducer and activator of transcription-3 (STAT3) signaling, along with secretion of proinflammatory cytokines (e.g., IL-17 and IL-23), thus accelerating cell proliferation and engendering tumorigenesis (48–51). Additionally, microbial composition is altered in patients with CAC or sporadic colorectal cancer and varies at different stages of colorectal tumorigenesis (52, 53), possibly owing to infection-associated inflammation, bacterial metabolites, and infection-induced oxidative stress (54). The changes of claudins in CAC and colorectal cancer are summed up in Table 1 (55–76).

**Table 1:** Changes of claudins in colitis-associated colorectal cancer (CAC) and colorectal cancer.

	**Human**		**Animal models**
	**CAC**	**Colorectal cancer**	
Claudin-1	↑(55, 56)	↑(57–60)↓(61, 62)	↓(63) (rats)
			↑(64) (mice)
Claudin-2	Unchanged (55)	↑(61, 65)↓(66)	Unchanged (63) (rats)
Claudin-3	↑(55)	↑(61, 67, 68)↓(58)	↓(69) (mice)
Claudin-4	↑(55)	↑(57, 67)↓(58, 62)	-
Claudin-5	-	↓(70)	-
Claudin-7	-	↑(61, 71)↓(58, 62, 72)	Unchanged (63) (rats)
		Unchanged (73)	↑(74)↓(75) (mice)
Claudin-8	-	↓(70, 76)	-
Claudin-12	-	↑(76)	-
Claudin-15	-	↓(70)	↑(63) (rats)

*Symbol “↑” stands for upregulation and “↑” for downregulation, while “-” stands for no explicit data on the respective claudin*.

## Expression and Function of Claudins

### Expression of Claudins in the Gastrointestinal Tract

Though claudins are widely expressed in various kinds of organs and tissues (77), only a few of them are detectable in the gastrointestinal tract. Multiple studies have confirmed the tissue-specific physiological heterogeneity in normal gastrointestinal epithelium and pathological modification of claudin expression in IBD patients (Table 2) (78–80). Hence, deviation from the physiological expression pattern of claudins probably implies a pathological state. The expression and distribution of claudins may be regarded as an indicator or mediator of mucosal function in concerning diseases that cause intestinal mucosal damage, including IBD.

**Table 2:** Expression patterns of claudins in normal human tissue in comparison with altered expression in IBD.

	**Normal tissue**						**Trend in IBD**	
	**Stomach**	**Duodenum**	**Jejunum**	**Ileum**	**Colon**	**Rectum**	**CD**	**UC**
Claudin-1	+(78)	+(78)	+(78, 79)	+(78)	+(78, 79)	+(78)	↑(78)	↑(78)
Claudin-2	+(78, 80)	+(78–80)	+(78, 79)	+(78–80)	±(78, 80)	±(78, 80)	↑(78)↓(80)	↑(78, 80)
Claudin-3	±(80)	±(79, 80)	No data	+(80)	+(78–80)	+(80)	↓(78, 80)	−(80) ↓(78)
Claudin-4	±(80)	+(79, 80)	No data	+(80)	+(78–80)	+(80)	↓(80)	−(80) ↓(78)
Claudin-5	No data	+(78, 79)	No data	No data	+(79)	No data	↓(78)	No data
Claudin-7	±(78, 80)	+(78, 80)	+(78, 79)	+(78–80)	+(78, 80)	+(78, 80)	No data	↓(78)
Claudin-8	±(80)	±(79, 80)	No data	±(80)	+(78–80)	+(80)	↓(78)	No data
Claudin-12	+(78, 80)	+(78, 80)	+(78, 79)	+(78, 80)	+(78, 80)	+(78, 80)	↓(78, 80)	−(80)
Claudin-15	±(78, 80)	+(78, 80)	+(78)	+(78, 80)	+(78, 80)	±(78, 80)	No data	No data
Claudin-18	+(80)	−(80)	No data	−(80)	−(80)	−(80)	No data	↑(78)

*The expression levels of claudins in normal human gastrointestinal tract were defined as positive (+), negative (−), and weakly positive (±). The trend of respective claudins in IBD was defined as upregulated (↑), downregulated (↓), and unchanged (−). IBD, Intestinal Bowel Diseases, UC, Ulcerative Colitis, CD, Crohn's Disease*.

### Structures of Claudins

**Figure 2:**
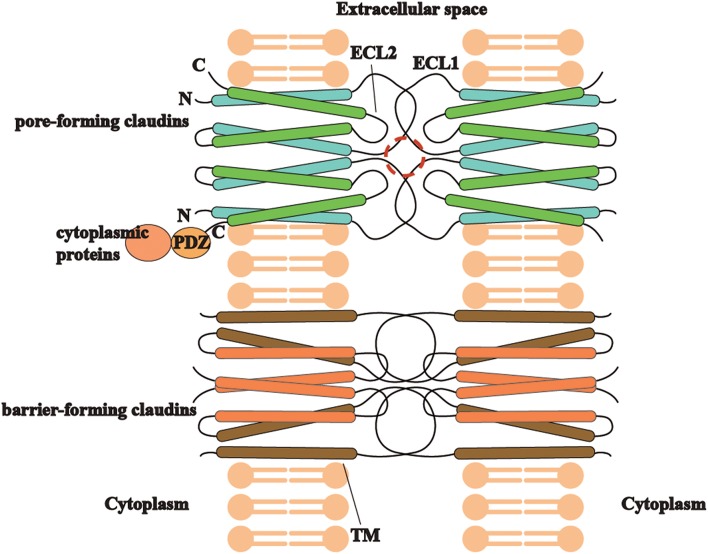
The trait of claudins (pore-forming or barrier-forming) depends on pore-like structures (marked in red circle) and polar amino acid residues of ECLs and occasionally TM segments in claudin dimers interaction. C-terminus of different claudins is diverse and able to bind cytoplasmic proteins by PDZ domain, which may be the structural foundation for claudins to modulate cell behaviors as signaling proteins.

Different types of claudins have basic structures in common (Figure 2), including four transmembrane helices, two extracellular loops, a short N-terminus and a cytoplasmic C-terminus(2). ECLs and TM mediate the formation of claudin dimers and dimers further assemble themselves into barrier or pores in the paracellular space (81, 82). ECLs are also able to bind *C. perfringens* enterotoxin and therefore to participate in infection-induced pathogenesis (82). On the other hand, the diverse C-terminus of claudins binds cytoplasmic proteins by PSD-95/Disc-large/ZO-1 (PDZ) domain, which may be the structural foundation for claudins to modulate cell behaviors as signaling proteins (83).

### Functions of Claudins in IBD and CAC

Though it is well-acknowledged that gastrointestinal-specific claudins participate in building intestinal barriers and modulating permeability (79), there is also ample evidence that claudins may act as signaling proteins and participate in inflammation, cell proliferation, differentiation, and tumorigenesis via cellular signaling pathways, including EGFR/MEK/ERK signaling, PI3K/Akt signaling, Wnt/β-catenin signaling and AMPK signaling, etc. (61). Thus, inflammation-induced claudins dysregulation may mediate the interaction between IBD and CAC.

#### Claudin-1

Claudin-1 is located in the junctional areas as well as lateral membranes of crypt epithelial cells and upregulated in entire epithelia during CD and UC (84, 85). Evidence shows that downregulation of claudin-1 contributes to increased intestinal permeability via NF-κB activation and leads to diarrhea in patients with irritable bowel syndrome (86). On the other hand, claudin-1-overexpressing mice exhibit higher susceptibility to experimental colitis, with impaired goblet cell differentiation, deferred epithelial recovery, sustained inflammation and crypt hyperplasia, possibly resulting from matrix metalloproteinases-9(MMP-9)/ERK-induced Notch signaling activation (85). Wnt/β-catenin signaling that participates in inflammation, cell differentiation and proliferation, seems to have a strong correlation with claudin-1, as claudin-1-overexpressing mice exhibit Wnt/β-catenin signaling activation, and caudal homeobox protein-2(Cdx-2)-associated Wnt signaling upregulates claudin-1 inversely in colorectal cancer cell lines (60, 64), along with combined upregulation of claudin-1 and β-catenin in human primary and metastatic colorectal cancer lesions (87). In addition, TNF-α-induced claudin-1 upregulation leads to ERK and SRC signaling activation, thus contributing to EMT and increased tumor invasion, suggesting the underlying role of claudin-1 in triggering colitis and CAC (88).

#### Claudin-2

As claudin-2 is predominantly expressed in TJ region and the apical cytoplasm of surface colonocytes (84), it is considered to increase paracellular permeability by forming cation-selective and water channels on intestinal epithelia (89, 90), along with an overall upregulation in IBD (84, 91) and CAC (92). Contrary to claudin-1, claudin-2-overexpressing mice exhibit higher resistance to experimental colitis by showing decreased cell apoptosis, increased epithelial proliferation and immune tolerance, brought about by downregulation of IL-6-induced NF-κB as well as STAT3 signaling and upregulation of T_reg_ cell population as well as immunoregulatory cytokine TGF-β (93). However, different from the protective role in colitis, claudin-2 is upregulated by Cdx-associated Wnt signaling activation (94) and contributes to tumorigenicity of CAC by promoting cell proliferation via EGFR/ERK signaling *in vitro* (92).

#### Claudin-3

Claudin-3 is typically located in junctional areas and lateral membranes of healthy colonic surface and crypt epithelia, while its expression in apical regions is lowered in the epithelia of IBD patients (78, 95). A research conducted on murine postnatal intestinal barrier development revealed that intestinal claudin-3 expression peaks during early stages of life and complies to the process of probiotics-induced MyD88-dependent gut maturation, indicating that claudin-3 forms intestinal epithelial barrier on the basis of probiotics-induced TLR activation in early life (96). Evidence from *in vitro* experiments also demonstrated that claudin-3 is downregulated by proinflammatory cytokines (e.g., IFN-γ, TNF-α, and IL-1β)-induced myosin light chain kinase (MLCK) activation (97, 98).In claudin-3-deficient mice model, colonic epithelial dedifferentiation, barrier dysfunction and rapid tumor progression likely resulted from IL-6/gp130/STAT3 signaling-induced Wnt/β-catenin signaling upregulation, indicating the interaction between inflammation and tumorigenesis mediated by claudin-3 deficiency (69). However, another study on colorectal adenocarcinoma cell line revealed that claudin-3 overexpression promotes tumor malignancy via EGFR/MEK/ERK and PI3K/Akt signaling (68). The aforementioned dual role of claudin-3 in tumorigenesis indicates that the imbalance of claudin-3 may participate in tumorigenesis via multiple pathways.

#### Claudin-4

Claudin-4 is expressed in TJs and lateral membranes of normal colonic surface as well as crypt epithelia, and responsible for physiological chloride reabsorption (95, 99). Downregulation of claudin-4 are mainly observed in junctional areas of inflamed epithelia, probably due to TNF-α/IFN-γ induced MLCK activation (95). AMPK and muscarinic acetylcholine receptors-induced ERK activation is also able to trigger claudin-4 phosphorylation, β-arrestin2/clathrin-mediated redistribution from cellular membranes to cytoplasm, and consequent ubiquitination-induced degradation (100, 101), contributing to increased epithelial paracellular permeability. However, studies on gastrointestinal tumor progression are quite contradictory. Although claudin-4 expression promoted by lncRNA-KRTAP5-AS1 and lncRNA-TUBB2A contributes to aggressive cancer behaviors in gastric cancer (102) and β-catenin expression is synchronous with claudin-4 upregulation in CAC tissues (55), there is also evidence showing that claudin-4 suppression in colorectal cancer tissues correlates with aggressive cancer behaviors (103).

#### Claudin-5

Claudin-5 localizes strictly in junctional areas in normal surface and crypt epithelia, whereas its reduction and redistribution from TJs to sub-junctional membranes are observed in active CD (91). Alteration in the expression of claudin-5 and other sealing claudins leads to decreased number of TJ strands and reduced depth of TJ meshwork, consequently increasing barrier permeability (91). Claudin-5 is upregulated by lamina propria lymphocytes for its function in accelerating intestinal epithelia differentiation via Notch-1 signaling to maintain epithelial homeostasis (104). However, the lymphoepithelial interactions may be a double-edged sword, as PI3K/Akt and MAPK signaling upregulated by lamina propria lymphocytes participate in dysregulated epithelial cell maturation and enhanced intestinal antigen presentation, therefore leading to exacerbation of inflammation (105). On the other hand, as claudin-5 is downregulated by vascular endothelial growth factor receptor-2 (VEGFR2)/PI3K/Akt signaling and IL-8 in endothelial cells, resulting in impaired endothelial integrity and increased vascular permeability (106), the reduction of claudin-5 may play a role in weakened cell adhesion and tumor metastasis in various tumor types, such as ovarian cancer (107), urothelial carcinoma (108), and prostate cancer (109). However, to date, the role of claudin-5 in gastrointestinal tumors remains obscure.

#### Claudin-7

Claudin-7 is found in TJ regions as well as basolateral membranes of colonocytes, whose junctional expression are significantly reduced in active UC (110). Deficiency of claudin-7 mainly causes dysregulated paracellular flux of small organic solutes, such as microflora products, and triggers colonic inflammation in mice models (111). In another study, due to claudin*-*7 deficiency, upregulation of MMPs (e.g., MMP-3 and MMP-7) turns IL-1β precursor into the active form (112) and triggers NF-κB signaling activation, along with MMPs-induced degradation of claudin-7/claudin-1/integrin-α2 complex, finally engendering colonic inflammation and damaged cell-matrix interaction (113). Further, claudin-7 inhibits ERK and SRC signaling, therefore suppressing EMT and tumor progression in colorectal cancer cell lines (114). However, there is also *in vitro* evidence that claudin-7 cooperates with epithelial cell adhesion molecule (EpCAM) and generates EpIC, a co-transcription factor that collaborates with β-catenin in cancer initiating cells, contributing to EMT and cancer metastasis (71).

#### Claudin-8

Since claudin-8 acts as sealing claudins in junctional regions of normal epithelial surface as well as crypts(91) and prevents paracellular back-leakage of Na^+^ in colonic reabsorption(115), its reduction and redistribution from TJs to cytoplasm may lead to TJ structure disruption and increased epithelial permeability in IBD, especially in active CD. In both IBD patients and mice with experimental colitis, claudin-8 is downregulated by hyperactivation of IL-23/miR-223 (116) and IL-9/miR21 (117) pathways, causing inflammation-induced intestinal mucosal damage and retardation of mucosal healing, while IL-9/STAT5 pathway activation may also account for bowel epithelial inflammation (118). On the other hand, as *CLDN8* is correlated with cancer progression via Akt and MAPK signaling in prostate cancer (119), the significance of downregulated claudin-8 in colorectal cancer awaits further analysis (76).

#### Claudin-12,-15, and -18

The presence of claudin-12 is decreased in the colon epithelia, but increased in the ileum epithelia of CD patients (80). By forming Ca^2+^ paracellular channels, claudin-12 mediates Vitamin-D dependent Ca^2+^ permeability in enterocytes (120). Although IL-18/p38 MAPK pathway downregulates claudin-12 and promotes tumor invasion in breast cancer, indicating its possible anti-tumor effect (121), the upregulation of claudin-12 in colorectal cancer needs to be further studied (76).

Claudin-15 modulates small intestinal Na^+^ permeability by forming Na^+^ channels (122), whose deficiency results in Na^+^-dependent glucose/amino acid/fat absorption defect in mice models (123, 124). Interestingly, claudin-15-deficient mice develop dilated and lengthened upper small intestines without signs of epithelial neoplasia, suggesting that claudin-15 may regulate intestinal epithelial cell proliferation and organ size, while the underlying mechanism remains to be discovered (125). Owing to a scarcity of research, the significance of reduced claudin-15 expression in colorectal adenocarcinoma is still undefined (70).

*CLDN18* gene produces lung-specific and stomach-specific claudin-18 (126). Stomach-specific claudin-18 (claudin-18.2) forms TJ strands in gastric epithelial cells and limits paracellular H^+^ efflux; its deficiency may trigger gastritis by upregulating IL-1β, TNF-α, and cyclooxygenase-2 (COX-2)/prostaglandin-E pathway (127). Intriguingly, claudin-18.2-deficient mice develop gastric mucosal neoplasia, which is associated with the activation of multiple pathways, including CD44, ephrin (EFN)/ ephrin receptor (EPH) and yes-associated protein-1 (YAP1)/HIPPO signaling (128). However, the existence of claudin-18.2 in primary and metastatic lesions of gastric cancer, along with its significant ectopic expression in various types of cancers (e.g., pancreatic adenocarcinomas, esophageal tumors, and bile duct cancers), indicates the underlying role of claudin-18.2 as a pan-cancer therapeutic target (129, 130). On the other hand, though lung-specific claudin-18 (claudin-18.1) is expressed in the colonic epithelia of UC patients and experimental colitis mice models, its significance awaits further analysis (131).

## Conclusion

Though claudins participate in the pathogenesis of IBD and consequent tumorigenesis, actual prognostic or therapeutic application of claudins in IBD remains scarce. As for the potential role of claudins in colorectal cancer, anti-claudin-1 antibodies specifically bind claudin-1 on the membrane of tumor cells and inhibit tumor progression *in vivo* and *in vitro*, indicating that claudin-1 may be a therapeutic target for colorectal cancer, especially for subtypes with *KRAS* mutations and Wnt signaling activation (132). However, even though claudin-1 is upregulated in human colon cancer tissues when compared with adjacent normal epithelia, claudin-1 reduction is strongly correlated with poor prognosis, including high recurrence rate and poor survival, in tumor-node-metastasis (TNM) stage II colon cancer patients (133). On the other hand, in a phase IIb study on patients with claudin-18.2 positive gastric cancer or gastroesophageal junction adenocarcinoma, the combination of anti-claudin-18.2 antibodies claudiximab and first line chemotherapy improves response rate, progression-free survival, and overall survival in comparison with chemotherapy alone, suggesting the potential of claudin-18.2 as a therapeutic target for claudin-18.2-positive gastric cancer (134) and other cancer types with claudin-18.2 ectopic expression (e.g., pancreatic cancer) (129, 135).

Previous studies have shown that claudins modulate barrier function, inflammation and tumorigenesis in gastrointestinal tract, while their roles in genetic propensity, immune response apart from T cells activation and microbial dysbiosis still need to be further studied. Also, for several gastrointestinal claudins (e.g., claudin−12,−15, and−18), where does the dysregulation exactly occur among various types of gastrointestinal cells and the relevant modulating mechanisms remain obscure. Though claudins dysregulation predominantly occurs in epithelial cells, whether it influences other cell types (e.g., intestinal stem cells and goblet cells) awaits further analysis. In addition, the sophisticated modulating mechanism of claudins makes it difficult to determine which signaling pathway is primary in IBD and CAC, especially for different cancer subtypes. Therefore, conclusions from different signaling studies may be contradictory, which has become an obstacle for claudins to be therapeutic targets for IBD and CAC. The potential of claudins in promoting mucosal healing by restoring TJ structures as TJ-forming proteins and regulating inflammation as well as tumor behaviors as signaling proteins awaits further analysis.

## Author Contributions

LZ and JH searched for and analyzed referenced articles. LZ and JH wrote the manuscript. LZ, JH, LL, YW, and YL summarized relevant information and designed the tables and figures in the review. SZ designed the study and revised the review. All authors agreed to be responsible for the content of the review.

### Conflict of Interest Statement

The authors declare that the research was conducted in the absence of any commercial or financial relationships that could be construed as a potential conflict of interest.

## Abbreviations

IBD: inflammatory bowel diseases
TJ: tight junction
CD: Crohn's Disease
UC: Ulcerative Colitis
CAC: colitis-associated colorectal cancer
TM: transmembrane
ECL: extracellular loop
EMT: epithelial-to-mesenchymal transition
ZO: Zonula Occludens
IL: interleukin
NF-κB: nuclear factor-κB
TLR: Toll-like receptor
IFN: interferon
PKC: protein kinase C
TGF-β: transforming growth factor-β
TNF: tumor necrosis factor
EGF: epidermal growth factor
AMPK: AMP-activated protein kinase
JNK: c-Jun N-terminal kinase
MAPK: mitogen-activated protein kinase
MEK: mitogen-activated ERK kinase
ERK: extracellular signal-regulated kinase
PI3K: phosphatidylinositol-3-kinase
Akt: protein kinase B
STAT: signal transducer and activator of transcription
PDZ: PSD-95/Disc-large/ZO-1
MMP: matrix metalloproteinases
Cdx: caudal homeobox protein
MLCK: myosin light chain kinase
VEGFR: vascular endothelial growth factor receptor
EpCAM: epithelial cell adhesion molecule
COX: cyclooxygenase
EFN: ephrin
EPH: ephrin receptor
YAP: yes-associated protein
TNM: tumor-node-metastasis.
